# Preliminary clinical study of left ventricular myocardial strain in patients with non-ischemic dilated cardiomyopathy by three-dimensional speckle tracking imaging

**DOI:** 10.1186/1476-7120-10-8

**Published:** 2012-03-07

**Authors:** Fengxia Duan, Mingxing Xie, Xinfang Wang, Yuman Li, Lin He, Lan Jiang, Qian Fu

**Affiliations:** 1 Department of Medical Ultrasound, Union Hospital, Tongji Medical College, Huazhong University of Science and Technology, Wuhan, People's Republic of China

**Keywords:** Echocardiography, Dilated cardiomyopathy, Ventricular function, left, Three dimensional speckle-tracking imaging

## Abstract

**Background:**

Non-ischemic dilated cardiomyopathy (DCM) is the most common cardiomyopathy worldwide, with significant mortality. Correct evaluation of the patient's myocardial function has important clinical significance in the diagnosis, therapeutic effect assessment and prognosis in non-ischemic DCM patients. This study evaluated the feasibility of three-dimensional speckle tracking imaging (3D-STE) for assessment of the left ventricular myocardial strain in patients with non-ischemic dilated cardiomyopathy (DCM).

**Methods:**

Apical full-volume images were acquired from 65 patients with non-ischemic DCM (DCM group) and 59 age-matched normal controls (NC group), respectively. The following parameters were measured by 3D-STE: the peak systolic radial strain (RS), circumferential strain (CS), longitudinal strain (LS) of each segment. Then all the parameters were compared between the two groups.

**Results:**

The peak systolic strain in different planes had certain regularities in normal groups, radial strain (RS) was the largest in the mid region, the smallest in the apical region, while circumferential strain (CS) and longitudinal strain (LS) increased from the basal to the apical region. In contrast, the regularity could not be applied to the DCM group. RS, CS, LS were significantly decreased in DCM group as compared with NC group (*P *< 0.001 for all). The interobserver, intraobserver and test-retest reliability were acceptable.

**Conclusions:**

3D-STE is a reliable tool for evaluation of left ventricular myocardial strain in patients with non-ischemic DCM, with huge advantage in clinical application.

## Background

Non-ischemic dilated cardiomyopathy (DCM) is the most common cardiomyopathy worldwide, with significant mortality. In this disorder, dilation and impaired contraction of the left or both ventricles develops [1]. DCM is characterized by dilated ventricles and systolic dysfunction, and the clinical symptom of non-ischemic DCM is heart failure, which is often associated with arrhythmia and sudden death [2,3]. Correct evaluation of the patient's myocardial function has important clinical significance in the diagnosis, therapeutic effect assessment and prognosis in non-ischemic DCM patients.

In recent years, the research on myocardial function in patients with non-ischemic DCM has become a hot topic in cardiovascular ultrasound [4-6], which mainly based on tissue Doppler imaging and two-dimensional speckle tracking imaging. However, the measurements of tissue Doppler imaging are angle dependent. Even though two-dimensional speckle tracking imaging solves this problem but it is also limited by a more difficult detection of LV movement, because it can not assess movement in the third dimension [7].

Three-dimensional speckle tracking imaging (3D-STE) technique is a new ultrasonographic method recently developed for noninvasive monitoring of regional and global myocardial function in clinical practice. Compared with the quantitative tissue velocity imaging and two-dimensional speckle tracking imaging, it has great advantages because it is independent of angle and does not ignore the characteristics of three-dimensional cardiac wall motion. Recently, a lot of studies have focused on different pathologic conditions [8-11], while the use of this novel technique in diagnosis of non-ischemic DCM is somewhat unclear. The aim of the present study was to evaluate the feasibility of 3D-STE for assessment of left ventricular systolic function in non-ischemic DCM patients.

## Methods

### Study population

Sixty-five outpatients and inpatients with non-ischemic DCM were consecutively enrolled in the study from Union Hospital, Wuhan. Eleven patients (16%) were excluded after echocardiographic acquisitions due to either irregular heart rhythm (n = 4) or insufficient image quality (n = 7). The remaining patients (36 males and 18 females), aged from 19 to 65 years old with an average of 48 years old were studied. The diagnosis of non-ischemic DCM was made on the basis of echocardiography (LV dilation and diffuse hypocontractility), electrocardiography (absence of Q waves), clinical criteria (no angina pectoris, no history of myocardial infarction), and coronary angiography (no significant coronary artery stenosis). Inclusion criteria included: (1)according with Framingham heart failure diagnostic criteria in 1971, NYHA classification ≥ IIgrade; (2) left ventricular ejection fraction (LVEF) < 50%; (3)LV end-diastolic dimension > 112% of the predicted value, corrected for age and body surface area. Exclusion criteria were presence of rhythms other than sinus rhythms in electrocardiography (ECG), pacemaker implantation, coronary artery disease (obstruction > 50% of the luminal diameter in a major branch), valvular heart diseases, and congenital heart diseases.

Fifty-nine healthy volunteers were consecutively enrolled as the control group (NC group), and seven subjects (11%) were excluded from this study because of insufficient image quality. The remaining 52 subjects (35 males, age 49 ± 12 years) with no organic disease based on physical examination, electrocardiogram, and echocardiography; and no risk factors for hypertension or coronary heart disease were finally included in the statistical analysis.

Each study was assessed for image quality using a four-grade scale based on the adequacy of visualization of LV segments. Image quality was graded as follows: good if 0-1 segments were poorly visualized; moderate if 2-3 segments were poorly visualized; poor if 4-5 segments were poorly visualized; and insufficient if > 5 segments were poorly visualized or if image quality was significantly compromised due to other factors. In DCM group, image quality was graded in the remaining 61 patients as insufficient in 7 (11%), poor in 9 (14%), moderate in 23 (37%), and good in 22 (36%). In NC group, image quality was graded in 59 subjects as insufficient in 7 (11%), poor in 9 (15%), moderate in 19 (32%), and good in 24 (39%).

### Conventional echocardiography

All the subjects were examined in the left lateral position, and ECG were recorded simultaneously. In this section, Artida PST-30SBT(1-5 MHz) phased-array transducer was used. After standard echocardiographic studies, in which end-diastolic diameters, end-systolic and end-diastolic volumes and ejection fractions were assessed using the biplane method according to the modified Simpson's rule, informed consents were obtained from those patients eligible for inclusion.

### Image acquisition

3DT was performed with Artida PST-25SX transducer (1-4 MHz) by one experienced operator. Apical full volume acquisition was obtained in all subjects to visualize the entire LV in a volumetric image within one breath hold. For acquisitions of a full-volume data set, six smaller real-time volumes, acquired from consecutive cardiac cycles, are combined to provide a larger pyramidal volume. Care was taken to optimize the temporal and spatial resolution of images by decreasing depth and sector width as much as possible while retaining the entire LV within the pyramidal volume. In this study, the frame rate was (21 ± 3) volumes/s.

### Three-dimensional speckle-tracking analysis

Three-dimensional speckle-tracking analysis was performed by one experienced observer on-line. Each 3D data set was displayed in a 5-plane view: (A) an apical 4-chamber view; (B) a second apical view orthogonal to plane A; and (C) 3 short-axis planes: plane C1 in the apical region, plane C2 in the midventricle, and plane C3 at the basal portion of the left ventricle. The user then set three markers on planes A and B; in each plane, one marker was set at the apex and the other two at the edges of the mitral valve ring. The software then detected the LV endocardium, and the user set a default thickness for the myocardium. After the markers had been selected, the system performed the wall motion-tracking analysis through the entire cardiac cycle. For each consecutive time frame, LV volume is calculated by voxel count inside the detected endocardial border. End-diastolic volume (EDV) and end-systolic volume (ESV) were then obtained from the LV volume curve as the respective maximum and minimum values, concurrently providing the calculated LVEF. The LV was divided into 16 segments (6 basal, 6 mid-LV, and 4 apical) on the basis of the American Heart Association standards for myocardial segmentation [12], and each segment was individually analyzed. The results of the 3D-WMT analysis were presented to the user as averaged values for each segment. The user was able to adjust the results of the tracking process when needed.

### Statistical analysis

Statistical analysis was carried out using SPSS version 13.0. Quantitative data were expressed as mean ± SD. Independent-samples *t *test was used for comparison between DCM group with NC group. Multiple comparisons among different planes were performed using analysis of variance (ANOVA) with post hoc SNK. Interobserver variability was assessed by analyzing 10 subjects chosen randomly by 2 independent investigators. For intraobserver variability, 10 subjects were analyzed by one investigator twice within 4 weeks, and the mean of the sixteen segments for RS, CS and LS were calculated, respectively. To assess test-retest reliability, a complete echocardiographic re-study was performed in 20 randomly selected subjects within 1 h after the first study without alteration of haemodynamics or therapy. Each study was subsequently analysed by different observers to best reflect daily clinical practice. The estimation of interobserver, intraobserver and test-retest reproducibility was performed using the intraclass correlation coefficient (ICC) and Bland-Altman plots. Pearson linear correlation method was used to analyze the correlation between 2D-derived and 3D-derived LV measurements. A *P *value less than 0.05 was considered to be statistically significant.

## Results

### Conventional echocardiography

Age, gender, BSA and heart rate showed no significant difference as compared with NC group (*P *> 0.05), while LVDd, LVEDV, LVESV, LVEF, LVEDVI, and LVESVI decreased significantly in DCM group (*P *< 0.01, Table 1).

**Table 1:** Conventional parameters of study subjects

Parameter	NC (n = 52)	DCM (n = 54)	*P *Value
Sex(male/female)	35/17	36/18	NS
Age(years)	47 ± 14	49 ± 11	NS
HR(/min)	72.63 ± 5.01	72.58 ± 5.30	NS
BSA(m^2^)	1.66 ± 0.11	1.69 ± 0.08	NS
LVDd(cm)	4.49 ± 0.42	7.13 ± 1.01	< 0.001
3D-LVEDV(ml)	81.37 ± 14.71	236.43 ± 84.26	< 0.001
2D-LVEDV(ml)	87.62 ± 16.12	241.82 ± 81.35	< 0.001
3D-LVESV(ml)	30.77 ± 6.07	169.60 ± 71.40	< 0.001
2D-LVESV(ml)	31.51 ± 6.41	172.23 ± 68.55	< 0.001
3D-LVEF(ml)	62.31 ± 2.56	30.87 ± 8.41	< 0.001
2D-LVEF(ml)	64.16 ± 2.59	33.77 ± 8.51	< 0.001
3D-LVEDVI(ml/m^2^)	48.84 ± 7.89	138.69 ± 47.93	< 0.001
3D-LVESVI(ml/m^2^)	18.44 ± 3.31	99.48 ± 41.00	< 0.001

Values given as mean ± SD. NS = not significant

LVDd = left ventricular end-diastolic diameter; BSA = body surface area; LVEDV = left ventricular end-diastolic volume; LVESV = left ventricular end-systolic volume; LVEF = left ventricular ejection fraction; LVEDVI = LVEDV/BSA; LVESVI = LVESV/BSA

### 2D-derived and 3D-derived LV measurements

The correlation of these two methods was good. Correlation coefficient for LVEDV, LVESV and LVEF were 0.926, 0.941 and 0.899 in DCM group, 0.965, 0.853, and 0.928 in NC group, respectively.

### Comparison of left ventricular regional strain curves and strain values

**Figure 1:**
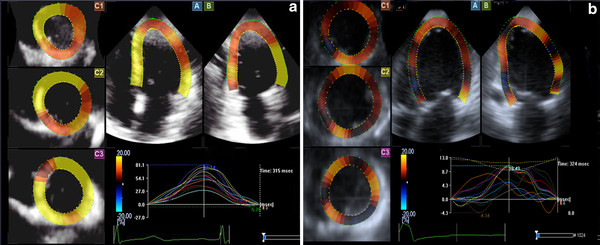
**Radial strain curves**. **A**: the radial strain curves of the normal group; **B**: the radial strain curves of DCM group.

**Figure 2:**
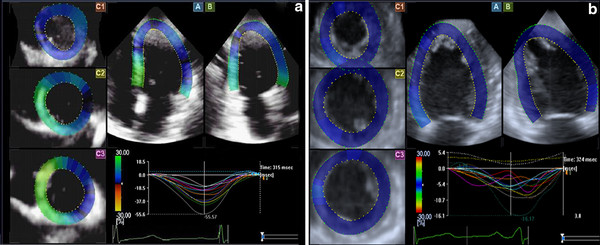
**Circumferential strain curves**. **A**: the circumferential strain curves of the normal group; **B**: the circumferential strain curves of DCM group.

**Figure 3:**
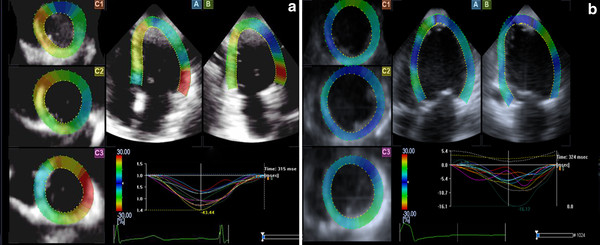
**Longitudinal strain curves**. **A**: the longitudinal strain curves of the normal group; **B**: the longitudinal strain curves of CHF group.

Normal regional strain curves showed consistent shapes with a single peak, which went to the peak around the end of the systolic phase. In contrast, the curve of DCM group was disordered, with delaying of the peak time (Figures 1, 2, 3).

RS, CS and LS of each segment were significantly decreased in DCM group as compared with NC group (*P *< 0.001 for all, Tables 2, 3, 4).

**Table 2:** Peak systolic radial strain values (%)

Parameter	NC (n = 52)	DCM (n = 54)	*P *Value
BA	34.1 ± 6.8	13.4 ± 5.3	< 0.001
BAS	34.6 ± 9.1	9.4 ± 3.7	< 0.001
BS	30.9 ± 6.5	10.5 ± 4.7	< 0.001
BI	27.5 ± 7.8	10.0 ± 4.5	< 0.001
BP	32.9 ± 9.6	11.9 ± 4.7	< 0.001
BL	34.5 ± 7.8	13.8 ± 5.1	< 0.001
MA	44.3 ± 11.8	9.3 ± 4.1	< 0.001
MAS	36.9 ± 10.3	10.7 ± 3.6	< 0.001
MS	33.2 ± 8.5	10.1 ± 4.7	< 0.001
MI	29.6 ± 8.3	9.4 ± 4.1	< 0.001
MP	36.3 ± 9.5	9.3 ± 4.3	< 0.001
ML	43.6 ± 13.3	8.4 ± 3.8	< 0.001
AA	29.2 ± 9.4	9.1 ± 4.3	< 0.001
AS	28.2 ± 7.4	8.8 ± 4.1	< 0.001
AI	25.5 ± 7.6	9.4 ± 3.7	< 0.001
AL	27.6 ± 8.6	6.6 ± 3.4	< 0.001

BA = basal anterior; BAS = basal anteroseptal; BS = basal inferoseptal; BI = basal inferior; BP = basal posterior; BL = basal lateral; MA = mid anterior; MAS = mid anteroseptal; MS = mid inferoseptal; MI = mid inferoseptal; MP = basal posterior; ML = basal lateral; AA = apical anterior; AS = apical septal; AI = apical inferior; AL = apical lateral

**Table 3:** Peak systolic circumferential strain values (%)

Parameter	NC (n = 52)	DCM (n = 54)	*P *Value
BA	-21.5 ± 5.4	-10.7 ± 3.2	< 0.001
BAS	-21.3 ± 4.6	-8.4 ± 3.4	< 0.001
BS	-27.8 ± 6.5	-9.8 ± 4.3	< 0.001
BI	-28.0 ± 5.5	-10.7 ± 3.3	< 0.001
BP	-27.8 ± 6.4	-12.6 ± 5.4	< 0.001
BL	-24.1 ± 9.6	-13.3 ± 5.1	< 0.001
MA	-24.5 ± 8.3	-9.5 ± 3.6	< 0.001
MAS	-25.1 ± 6.7	-10.6 ± 4.8	< 0.001
MS	-33.3 ± 7.8	-11.9 ± 4.4	< 0.001
MI	-32.6 ± 4.4	-10.9 ± 4.2	< 0.001
MP	-31.4 ± 5.3	-10.9 ± 4.2	< 0.001
ML	-25.3 ± 7.4	-9.6 ± 4.2	< 0.001
AA	-30.4 ± 9.2	-9.4 ± 4.8	< 0.001
AS	-37.5 ± 11.8	-10.2 ± 5.4	< 0.001
AI	-34.6 ± 10.4	-10.9 ± 5.5	< 0.001
AL	-29.2 ± 8.2	-9.8 ± 4.2	< 0.001

BA = basal anterior; BAS = basal anteroseptal; BS = basal inferoseptal; BI = basal inferior; BP = basal posterior; BL = basal lateral; MA = mid anterior; MAS = mid anteroseptal; MS = mid inferoseptal; MI = mid inferoseptal; MP = basal posterior; ML = basal lateral; AA = apical anterior; AS = apical septal; AI = apical inferior; AL = apical lateral

**Table 4:** Peak systolic longitudinal strain values (%)

Parameter	NC (n = 52)	DCM (n = 54)	*P *Value
BA	-13.4 ± 4.2	-9.3 ± 4.1	< 0.001
BAS	-16.3 ± 4.1	-6.7 ± 3.3	< 0.001
BS	-12.4 ± 3.5	-6.2 ± 2.5	< 0.001
BI	-16.3 ± 4.2	-8.2 ± 3.2	< 0.001
BP	-15.4 ± 4.5	-10.5 ± 3.5	< 0.001
BL	-14.9 ± 6.4	-10.3 ± 3.2	< 0.001
MA	-14.2 ± 5.5	-7.2 ± 3.5	< 0.001
MAS	-17.3 ± 4.2	-7.1 ± 3.2	< 0.001
MS	-18.1 ± 3.4	-7.4 ± 3.2	< 0.001
MI	-17.2 ± 4.4	-6.9 ± 2.6	< 0.001
MP	-19.3 ± 6.3	-6.6 ± 3.2	< 0.001
ML	-16.9 ± 4.7	-6.4 ± 2.6	< 0.001
AA	-22.3 ± 5.1	-7.8 ± 3.5	< 0.001
AS	-28.2 ± 6.4	-10.3 ± 3.2	< 0.001
AI	-27.4 ± 6.0	-8.6 ± 3.4	< 0.001
AL	-20.4 ± 5.2	-7.6 ± 3.2	< 0.001

BA = basal anterior; BAS = basal anteroseptal; BS = basal inferoseptal; BI = basal inferior; BP = basal posterior; BL = basal lateral; MA = mid anterior; MAS = mid anteroseptal; MS = mid inferoseptal; MI = mid inferoseptal; MP = basal posterior; ML = basal lateral; AA = apical anterior; AS = apical septal; AI = apical inferior; AL = apical lateral

### Comparison of myocardial strain from basal to apical region

The peak systolic strain in different planes had certain regularities in NC group, RS was the largest in the mid region and smallest in the apical region, while CS and LS were the largest in the apical region and smallest in the basal region (*P *< 0.05). Whereas the regularity could not be applied to the DCM group, as there were no significant differences between any two planes (*P *> 0.05, Table 5).

**Table 5:** Differences in myocardial strain from basal to apical region (%)

	Base	Mid	Apex
Radial strain			
NC(n = 52)	33.34 ± 7.65	38.57 ± 8.59^a^	27.58 ± 9.74^a,b^
DCM(n = 54)	11.63 ± 4.86	9.82 ± 4.85	8.62 ± 4.49
Circumferential strain			
NC(n = 52)	-24.85 ± 5.29	-28.72 ± 5.62^a^	-32.47 ± 10.73^a,b^
DCM(n = 54)	-11.35 ± 3.62	-10.08 ± 4.39	-9.87 ± 5.47
Longitudinal strain			
NC(n = 52)	-14.39 ± 3.29	-17.81 ± 3.16^a^	-24.85 ± 5.81^a,b^
DCM(n = 54)	-8.61 ± 2.84	-6.92 ± 3.09	-8.73 ± 3.19

^a^*P *< 0.05 vs basal region; ^b^*P *< 0.05 vs mid region

### Reproducibility

**Figure 4:**
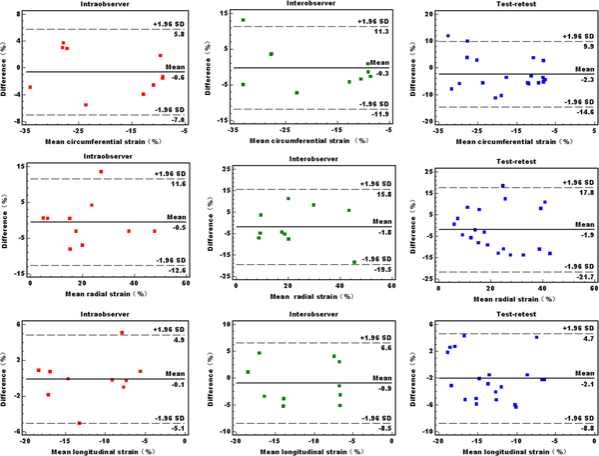
**Reproducibility of left ventricular strain measurements**. Bland-Altman plots for intra-observer (left), inter-observer (middle), and test-retest (right) reproducibility of left ventricular circumferential strain, radial strain and longitudinal strain.

Intraobserver, interobserver and test-retest reliability for RS, CS, and LS measurements on 3D-STE were acceptable. As expected, the intraobserver reliability was better than interobserver and test-retest reliability. ICCs were shown in Table 6, and results of Bland-Altman analyses of strain measurements were shown in Figure 4.

**Table 6:** ICCs of left ventricular strain measurements

Variable	Inraobserver	Interobserver	Test-retest
Circumferential strain	0.95	0.92	0.79
Radial strain	0.89	0.80	0.69
Longitudinal strain	0.87	0.85	0.69

Data expressed as intraclass correlation coefficient (ICC)

## Discussion

During the development of congestive heart failure (CHF) in patients with DCM, the left ventricle undergoes complex changes in geometry, by alteration of the left ventricular (LV) chamber and myocardial systolic and/or diastolic function. Determination of myocardial function is very important and necessary in the clinical evaluation of non-ischemic DCM. It not only assists in the diagnosis, but also provides important prognostic and management information. Nowadays, echocardiography is the imaging modality most widely utilized to assess left ventricular (LV) myocardial function, and the most widely used clinical tool to quantify LV systolic function is ejection fraction (EF), but it utilizes a simplistic approach that correlates most closely to the radial performance of the myocardium. Strain and Strain Rate imaging allow for a more precise characterization of the mechanics of myocardial contraction and relaxation (deformation imaging).

Strain can be determined by either tissue Doppler or two-dimensional speckle tracking. The prior method is influenced by the angle between the direction of ultrasound beam and myocardial motion [13]. Therefore, the tissue Doppler imaging is mainly used to detect one-dimensional myocardial motion and strain in the long axis parallel to ultrasound beam [14]. Unlike Doppler derived strain, Two-dimensional speckle tracking (2D-STE) is angle independent, which measures the strain by tracking speckles, which are acoustic backscatter generated by ultrasound interactions with the myocardium [15]. However, 2D-STE has limitations for its 2D imaging-based method. The out-of-plane problem inherent in short-axis imaging is caused by longitudinal heart motion during the cardiac cycle. In addition, circumferential rotation also contributes to 3D wall deformations and affects tracking accuracy [9,16]. More recently, three-dimensional speckle-tracking (3D-STE) was developed [17], allowing more complete and accurate assessment of myocardial deformation in 3D space by avoiding the loss of speckles due to out-of-plane motion [18]. Actually the cardiac movement is three-dimensional. What 2D-STE tracked is the projection of 3D movement on the two-dimensional plane, whereas 3D tracking assesses real movement in 3D space. 3D-STE is not based on regions of interest; instead, it is performed on the complete LV myocardium in the 3D data set. Many thousands of speckles are tracked in this volume [7]. The results of the wall motion tracking are based on these thousands of vectors. 3D-STE combines the usefulness of wall motion tracking with a better integration of heart anatomic structures. Furthermore, it may assess a higher proportion of myocardial segments and is able to acquire and analyze 3D dataset consuming less time [19]. To date, its usefulness has been demonstrated for the quantification of LV volumes and function (both global and regional), the dyssynchrony and rotation [8,19-23]. Pérez de Isla [7] and Saito [24] compared 2D-STE with 3D-STE, showed that 3D-STE provided complete radial and longitudinal and circumferential LV strain information, similar to 2D-STE, with less time consuming and good interobserver and intraobserver agreement, and the percentage of segments analyzed with 3D-STE was greater than that with 2D-STE, Nesser [25] evaluated the accuracy of the new 3D-STE side by side with 2D-STE using cardiac magnetic resonance as a reference, validated the new 3D-STE technique for LV volume measurements and demonstrated its superior accuracy and reproducibility over previously used 2D-STE technique. In our work, the ICCs of RS, CS and LS were calculated, and the interobserver, intraobserver and test-retest reliability were acceptable. As expected, the intraobserver reliability was better than interobserver and test-retest reliability. In addition, 3D LV volumes and ejection fractions could be simultaneously provided together with 3D strain values, which had good correlation with 2D-derived LV measurements.

The orientation of the fibers in the intact LV wall are complicated, which change from an oblique orientation at the epicardium to a circumferential orientation at the midwall and then to a reverse oblique direction at the endocardium [26]. That is to say the fibers are right-handed helical in the subendocardial region and gradually changes into left handed helical in the subepicardial region, and the mid-wall fibres exhibiting an intermediary circumferential orientation. Therefore, myocardial deformation occurs in three dimensions and can be characterized not only in the longitudinal direction, but also in the circumferential and radial directions. Subepicardial fibers play a major role in radial strain and longitudinal strain [26], and subendocardial fibers take an important part in circumferential strain [27].

In the present study, all myocardial deformational parameters, including radial, circumferential and longitudinal strain were significantly lower in patients with DCM than in the control group, which was in agreement with previous reports of depressed contractility in patients with DCM [28,29]. A large number of studies have shown that the basic mechanism of the development of heart failure is ventricular remodeling [30]. The left ventricle dilated, from oval to spherical. This changed the running angle of myocardial fiber, from oblique to horizontal, especially in the apical myocardial fiber. In the process of cardiac enlargement, corresponding changes took place in the myocardial cells, extracellular matrix and collagen fiber network. At the same time, myocardial perfusion reduced, which increased subendocardial and subepicardial myocardial ischemia, which therefore, led to myocardial necrosis and fibrosis ultimately. Reduction of myocardial cells and myocardial fibrosis could lead to severe systolic cardiac dysfunction [31].

In our study, we found that normal regional strain curve showed consistent shapes with a single peak, and the peak systolic strain in different planes had certain regularities. CS and LS were largest in the apical region and smallest in the basal region, which was in accordance with previously reported works using 3D-STE and 2D-STE [24,32] and magnetic resonance imaging tagging [33]. RS was smallest in the apical region, which was concordant with some previous reports [24,32], but discordant with others [33,34]. This discordance may due to using different modalities and machines, such as 2D-STE, which has several limitations in accurate assessment of myocardial function. It may be also related to its larger susceptibility to differences in spatial resolution. In contrast, the curve of DCM group was disordered, and there were no significant differences between any two planes, which implied that left ventricular systolic function reduced diffusely in patients with DCM.

## Limitations

Our results should be considered in the context of several limitations. First, this study covered a small number of patients at a single center, so that further studies of larger patient populations will be needed. Second, time and spatial resolutions of 3D-STE are relatively low. Thus, the low frame rate of 3D-STE could cause miscorrelation between frames and possibly may have affected tracking quality and strain data. This is a usual limitation in ultrasound, and we are waiting for improvements.

Third, in our work, data on 3D-STE were obtained using a particular platform and a specific implementation of wall-motion-tracking software. It may not be assumed that identical data would be obtained using a different platform or a different tracking algorithm. In previous studies, the inter-vendor consistency of speckle-derived indices has been investigated [18,35], and 3D-STE-derived LV deformation parameters are highly vendor dependent. In our study, we used only one equipment, so the comparison with other vendor was needed. Finally, this is a preliminary clinical study, not a validation study. Because an independent reference technique is lacking, such as magnetic resonance imaging, our work represents a study of the feasibility of 3D-STE but does not establish the accuracy of the new method.

## Conclusions

The newly developed technique of 3D-STE is a reliable tool for evaluation of left ventricular myocardial strain in patients with non-ischemic DCM, and the interobserver and intraobserver agreement and test-retest reliability were acceptable. Furthermore, 3D LV volumes and ejection fractions were simultaneously provided together with 3D strain values, which is a huge advantage in clinical application.

## Competing interests

The authors declare that they have no competing interests.

## Authors' contributions

FXD and MXX have made substantial contributions to study conception and design, acquisition of data, analysis and interpretation of data. XFW, YML and LH have been involved in revising study critically and have given final approval of the version to be published. LJ and QF have been involved in acquisition and analysis of data, and given final approval of the version to be published. All authors read and approved the final manuscript.
